# Problem Gambling and Problem Gaming in Elite Athletes: a Literature Review

**DOI:** 10.1007/s11469-021-00692-y

**Published:** 2021-12-01

**Authors:** Anders Håkansson, N. Durand-Bush, G. Kenttä

**Affiliations:** 1 Dept. of Clinical Sciences Lund, Psychiatry, Faculty of Medicine, Lund University, Lund, Sweden; 2 Clinical Sports and Mental Health Unit, Malmö Addiction Center, Region Skåne, Malmö, Sweden; 3 Malmö Addiction Center, Region Skåne, Södra Förstadsgatan 35, plan 4. S-205 02, Malmö, Sweden; 4 School of Human Kinetics, University of Ottawa, Ottawa, Canada; 5 Canadian Centre for Mental Health and Sport, Orleans, Canada; 6 The Swedish School of Sport and Health Sciences, Stockholm, Sweden; 7 Swedish Sport Federation, Stockholm, Sweden

**Keywords:** Elite athlete, Gambling disorder, Gaming disorder, Behavioral addiction

## Abstract

Researchers have raised concerns about mental health in elite athletes, including problem gambling, where research hitherto is scarce. While gambling has been assessed in the younger student-athlete population, neither gambling nor the recently recognized behavioral addiction of gaming disorder has been sufficiently addressed in the elite athlete population. The present systematic literature review aimed to summarize research knowledge on the prevalence and correlates of problem gambling and problem gaming in elite athletes. Research papers were searched systematically using the Scopus, PsycINFO, and PubMed/MEDLINE databases and evaluated following a PRISMA paradigm. For the elite athlete population, eight reports on problem gambling and one report on problem gaming were found. While at least five papers indicated an increased risk of problem gambling in elite athletes compared to the general population, one study from Australia indicated the opposite. Problem gambling was generally more common in male athletes. Knowledge of problem gaming prevalence is thus far limited. It is concluded that increased research in problem gambling and problem gaming in elite athletes is warranted.

Mental health in high-performance athletes is a growing concern and has been highlighted by researchers during the past decade (Reardon & Factor, 2010; Reardon et al., 2019; Rice et al., 2016; Schinke et al., 2018). Overall, mental health in elite athletes may be challenged by contextual factors specific to the world of competitive sports, but it may also be affected by stigma, difficulties in treatment access, and by a risk of athletes being perceived as healthier than they are, due to their great physical capacity and seemingly high degree of personal success and life satisfaction. There has been increasing attention to the need for specialized interventions for elite athletes, such as specific treatment facilities, both due to the characteristics of mental health challenges in athletes and potential gaps in regular or traditional mental health care (Moesch et al., 2018; Van Slingerland et al., 2019).

Although the mental health issues seen in elite athletes and the general population include a broad range of disorders and symptoms, some behavioral addictions have been highlighted as a potential risk for athletes (Reardon et al., 2019; Rice et al., 2016). Among the addictive disorders studied in elite athletes is the gambling disorder. Problem gambling, including the narrower concept of the gambling disorder, is a well-established construct in diagnostic manuals (American Psychiatric Association, 2013; World Health Organization, 2018) and known to affect somewhere between less than 1% and up to 5–6% of the general population, across different settings and studies (Calado & Griffiths, 2016). A gambling disorder has severe consequences on the mental well-being of the affected person, including financial consequences (Reith et al., 2019), high psychiatric comorbidity (Dowling et al., 2015; Håkansson et al., 2018a), suicidal ideation (Ronzitti et al., 2016), and a statistically elevated risk of suicide death (Karlsson & Håkansson, 2018). Also, given the transfer to an increasing online-based gambling market (Chóliz, 2016), this presents further challenges with respect to increased gambling availability.

Over and above the importance of assessing problem gambling in the general population, gambling may present specific hazards to the elite athlete population. The link between gambling and the world of sports may seem intuitive and can be hazardous in a number of ways. Scholars have raised concerns about attitudes toward gambling among athletes, and the risk of match fixing has received growing attention during the past few years (Frenger et al., 2019), including the risk related to betting on one’s own game (Moriconi & de Cima, 2020). In addition, sport-related contexts are often exposed in gambling advertisements related to sports betting (Deans et al., 2017; Hing et al., 2017; Pitt et al., 2016).

A number of research papers over the past decades have reported gambling and gaming problems among student-athletes, typically college-athletes from the National Collegiate Athletic Association (NCAA) in the USA (Derevensky et al., 2019). However, in line with the increased attention on mental health in elite athletes (Reardon & Factor, 2010; Reardon et al., 2019; Rice et al., 2016; Schinke et al., 2018), it remains to be fully understood how prevalent problem gambling is among athletes at the elite level. Elite athletes are typically described as athletes in a professional role, or those who compete individually or in teams at a national or international competitive level, such as in the top leagues of team sports (Swann et al., 2015). Elite athletes may be role models for younger athletes and for the general public, and the extent of gambling problems in elite athletes, over and above other health concerns in affected individuals, may also influence attitudes toward gambling in the general population.

The research field of behavioral addictions has expanded, and while gambling disorder so far has represented the only non-substance addictive disorders recognized by diagnostic manuals, problems related to video game behavior have been increasingly highlighted and recently included as a diagnosis in the World Health Organization (2018) diagnostic system (ICD-11), named the *gaming disorder*. Likewise, few years prior to this, the corresponding disorder (*internet gaming disorder*) was addressed by the DSM-5 working group and included as a potential diagnosis in need for more research (American Psychiatric Association, 2013).

The extent of problem gaming in the general population is less established than for problem gambling. A large study in the Norwegian general population described that among individuals with any gaming behavior, 1.4% were classified as addicted and another 7.3% were classified as problem gamers (Wittek et al., 2016). Moreover, there is reason to believe that gambling for money and video gaming are behaviors which in many cases are inter-dependent, and problem gambling and problem gaming have been shown to be associated to a certain degree (Karlsson et al., 2019; Wood et al., 2004). Of note, relevant differences have also been identified between clinical patients with gambling disorder and gaming disorder, respectively (Mallorquí-Bagué et al., 2017). Given the novelty of the gaming disorder diagnosis, it is important to research its prevalence in the general population and in specific risk groups. The knowledge about problematic gaming practices among elite athletes are thus far anecdotal rather than based on systematic research data. Media reports have highlighted video gaming as a time-consuming habit in elite soccer players, which could potentially be problematic (Washington Post, 2018, 2020). Time-consuming travelling, for example, may be a factor stimulating increased online behavior including gaming. However, the prevalence and correlates of gaming disorder or problem gaming in elite athletes are largely unknown (Håkansson et al., 2018b).

Altogether, there is need for further research in this area to direct attention to sport-specific contexts and mental health in the population of competitive athletes (Moesch et al., 2018; Van Slingerland et al., 2019). Therefore, the present review aimed to summarize research describing the prevalence and correlates of problem gambling and problem gaming among elite athletes, with an emphasis on research clearly addressing high-performance athletes.

## Method

**Fig. 1:**
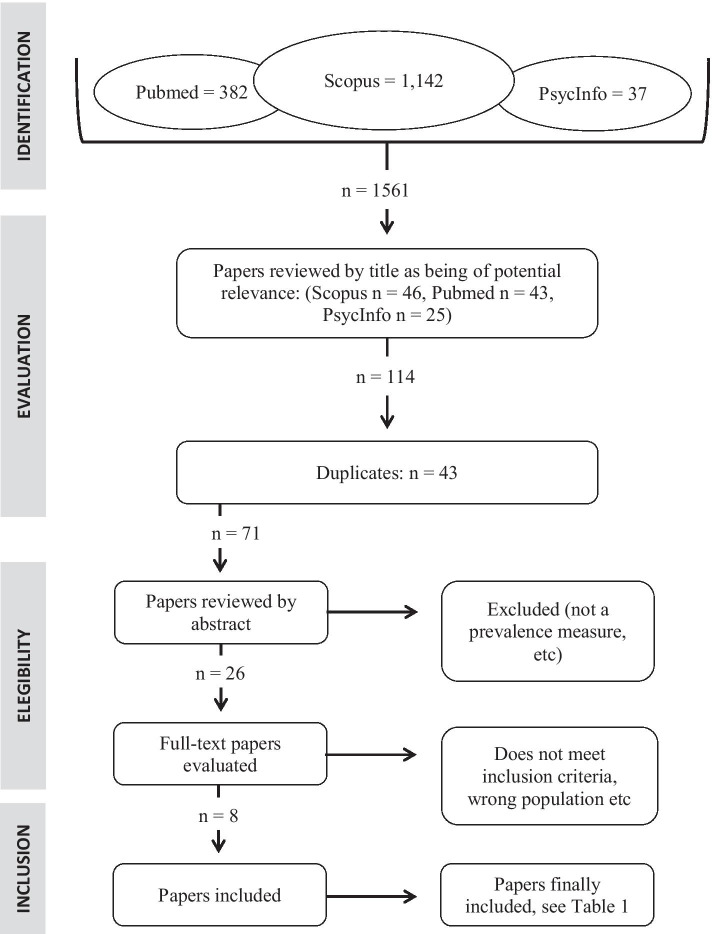
PRISMA flowchart assessing problem gambling and gambling disorder in athletes. Search words used: problem gambling OR gambling disorder AND athlete

**Fig. 2:**
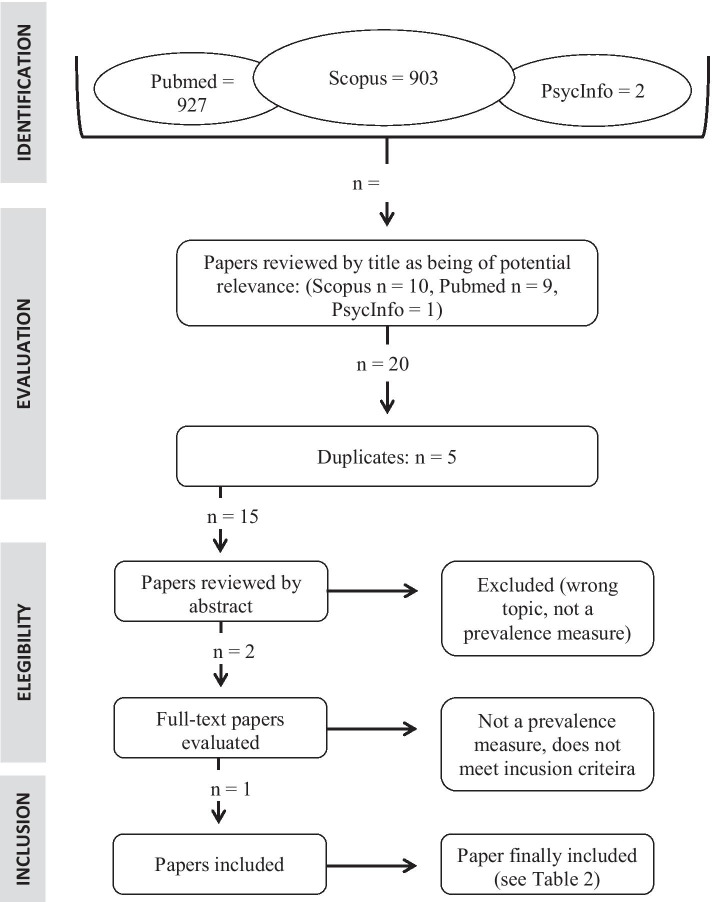
PRISMA flowchart assessing problem gaming and gaming disorder in athletes. Search words used: problem gaming OR gaming disorder AND athlete

The present study is a systematic literature review addressing the prevalence of problem gambling or gambling disorder and problem gaming and gaming disorder in elite athletes. The study followed a PRISMA paradigm for the literature search, selection of papers, and interpretation of findings (Figs. 1 and 2). The literature search was carried out by the first author.

### Eligibility Criteria

This systematic review study included papers using the following inclusion criteria: studies identified in the databases listed below which assess the prevalence of problem gambling, gambling disorder, problem gaming, or gaming disorder and which assess individuals specifically described as elite athletes. Papers in English, German, French, Spanish, Italian, or in any of the Scandinavian languages were considered. The literature search was carried out by the first author in July 2021. No restrictions were applied with respect to year of publication. Based on the literature and discussion with the research team, elite athletes were regarded as professional and/or perceived in the paper to represent national teams, top leagues, or a corresponding level (Swann et al., 2015). The major distinction made in the present review was between elite athletes and student-athletes/college-athletes who were in many cases enrolled in studies due to their involvement in the NCAA (Derevensky et al., 2019). Papers on student-athletes/college-athletes were reviewed with respect to the competitive level of study participants. None of these papers met the inclusion criteria used in the study regarding elite athletes and was therefore excluded from the review.

### Information Sources and Search Strategy

The following search strategy was applied within the literature search: the databases Scopus, PsycINFO, and PubMed/MEDLINE were searched for “gambling disorder” OR “problem gambling” AND “athlete” and “gaming disorder” OR “problem gaming” AND “athlete,” respectively. In addition, identified review papers were searched for potential further papers. In order to conduct a further control search aiming to identify any potentially relevant papers left out in the original searches, a control was carried out in the top relevance findings in the same searches, respectively, on Google Scholar. In addition to the papers identified through this literature search, one paper assessing gambling was identified from a reference list and added in the review.

### Selection of Papers and Data Collection Processes

The results of the literature search are seen in Table 1. A total of 57 papers (including one paper added from a reference list) regarding problem gambling and 24 papers regarding problem gaming were considered, but most papers failed to meet the inclusion criteria. After reading abstracts and—whenever needed—full-text versions, six papers with respect to problem gambling and one paper with respect to problem gaming were judged to fulfill the inclusion criteria. All papers that finally qualified for full-text reading were either in English or German.

**Table 1:** Studies included in the final review—gambling

Publication	Setting, number of individuals, gender, and sports distribution	Competitive level	Screening/diagnostic instrument used	Prevalence findings in men and women	Major findings of potential correlates
Rhind et al. (2014)	UK, athletes (*N* = 1,049), 53% male, large number of sports	Various levels, from international/national (5%) to county (32%) or club (63%) competitive level	Problem Gambling Severity Index, PGSI (Wynne & Ferris, 2001), past 12 months	Moderate-risk and problem gambling 13.7% and 9.2% in males, 1.5% and 1.1% in females	Higher than in general population both for men and women. Higher problem gambling in team sports (9.1% vs 3.1%, *p* < 0.01). No association with age, income, competitive level, etc
Grall-Bronnec et al. (2016)	Spain, France, Greece, Ireland, Italy, Sweden, UK (*N* = 1,236), several team ball sports	Professional athletes	Lie/Bet (Götestam et al., 2004; Johnson et al., 1997), lifetime	8.2% current or previous problem gambling	Associations with gambling online, betting regularly, betting online, and positive urgency
Håkansson et al. (2018b)	Sweden, athletes (*N* = 352), 60% female, large number of sports	National team level and conduct college/university (and corresponding) studies	NODS-CLiP (Toce-Gerstein et al., 2009; Volberg et al., 2011), lifetime	Problem gambling in 14% of males and 1% of females	Higher than in general population, at least in men. No difference in team/non-team sports, age, mental health treatment-seeking or hazardous alcohol consumption
Jensen et al. (2019)	Denmark and Sweden, soccer players (*N* = 323), 100% male	Elite soccer players, “top-level teams”	BBGS (Brief Biosocial Gambling Screen, Gebauer et al., 2010), past 12 months	16.1% problem (“at-risk”) gambling	Higher than in general population. Associated with depression and sport anxiety. No association with age
Vinberg et al. (2020)	Sweden, athletes (*N* = 1,438), 65% male, all team sports (soccer, ice hockey, floorball, basketball)	Top-three divisions for each sport	PGSI (Wynne & Ferris, 2001), past 12 months	Moderate-risk + problem gambling 13.1% in males and 1.7% in females	Clearly higher in males than in general population. No difference across employment status. No obvious differences between type of sport (significance levels not shown)
Purcell et al. (2020)	Australia, elite athletes (*N* = 749), 37 sports (61% individual), 52.5% male	Elite athletes (registered in high-performance system database for Olympic, Paralympic or Commonwealth game sports)	PGSI (Wynne & Ferris, 2001), past 12 months	94.0% non-problem gambling (i.e. up to 6% low-risk, moderate-risk or problem gambling)	Above non-problem gambling markedly less common than in the general population. Associations (including with gender) not reported
Håkansson et al. (2020b)	Sweden, elite athletes, top leagues of football, ice hockey, handball (*N* = 274), assessed during COVID-19	Top national leagues (highest female leagues, highest male handball league and highest two male football and ice hockey leagues)	PGSI (Wynne & Ferris, 2001), past 12 months	Moderate-risk/problem gambling in 10% of men, and 0% of women	Clear association of gambling problems with male gender
Pensgaard et al. (2021)	Norway, several types of sports, assessed during COVID-19 (June–September, 2020), (*N* = 378)	Olympic/Paralympic athletes and other elite/semielite athletes	Canadian Problem Gambling Index (CPGI, Wynne & Ferris, 2001)	“Gambling problems” in 8.7% of men and 1.3% of women	Gambling problems (definition unclear) markedly higher in males than in females, and lower in athletes reporting positive consequences of the COVID-19 situation

## Results

### Gambling Disorder and Problem Gambling

Among the eight papers related to gambling, seven were identified in peer-reviewed scientific journals in English (Grall-Bronnec et al., 2016; Håkansson et al., 2018b, 2020b; Jensen et al., 2019; Pensgaard et al., 2021; Purcell et al., 2020; Vinberg et al., 2020), whereas one publication was a book chapter (Rhind et al., 2014). The years of publication ranged from 2014 to 2021. Three of the studies were from Sweden (Håkansson et al., 2018b, 2020b; Vinberg et al., 2020), one from Australia (Purcell et al., 2020), one from the UK (Rhind et al., 2014), one from France but assessing participants from seven European countries (Grall-Bronnec et al., 2016), one from Sweden and Denmark combined (Jensen et al., 2019), and one from Norway (Pensgaard et al., 2021). One study involved only men (Jensen et al., 2019) and one involved primarily men (Grall-Bronnec et al., 2016), whereas the remaining six included a substantial number of both women and men. Four studies involved a number of different sports (Håkansson et al., 2018b; Pensgaard et al., 2021; Purcell et al., 2020; Rhind et al., 2014), three studies involved a variety of team sports (Grall-Bronnec et al., 2016; Håkansson et al., 2020b; Vinberg et al., 2020), and one study involved only soccer players (Jensen et al., 2019). All eight studies included validated measures of problem gambling (Table 1), and none reported the prevalence of an actual gambling disorder.

In the six studies assessing substantial percentages of participants from both genders, prevalence rates of problem gambling were concluded to be higher in men than in women (Håkansson et al., 2018b, 2020b; Pensgaard et al., 2021; Purcell, et al., 2020; Rhind et al., 2014; Vinberg et al., 2020). In five of the studies (Håkansson et al., 2018b, 2020b; Jensen et al., 2019; Rhind et al., 2014; Vinberg et al., 2020), authors specifically concluded that prevalence rates of problem gambling for men were higher than what would have been expected in the general population, whereas the opposite was seen in the Australian study by Purcell et al (2020). For the UK study (Rhind et al., 2014), it was concluded by the authors that problem gambling was also more common among women athletes than women in the general population, while these conclusions appeared less certain in the papers by Håkansson et al. (2018b), Håkansson et al. (2020b), and Vinberg et al (2020).

Three of the studies allowed comparison between different types of sports. In the UK study by Rhind et al. (2014), team sport participants were significantly more likely than individual athletes to be problem gamblers. In contrast, in the study by Håkansson et al., (2018b), no difference was seen between team sport athletes and individual sport athletes. In the Swedish study by Vinberg et al (2020), no statistical comparison across team sports was explicitly stated, but the reported prevalence rates were comparable across ice hockey, soccer, and basketball.

### Gaming Disorder and Problem Gaming

The sole paper addressing problem gaming (but not gaming disorder) was the same publication as one of those assessing problem gambling in elite athletes in Sweden, from a large number of different sports. The study demonstrated that past-6-month problem gaming was seen in 4% of males and 1% of females, with a trend toward a significant difference, and problem gaming was significantly associated with problem gambling (Table 2). However, the association between problem gambling and the absolute value of the screening tool used for gaming did not reach statistical significance (Håkansson et al., 2018b).

**Table 2:** Studies included in the final review—gaming

Publication	Setting, number of individuals, gender, and sports distribution	Competitive level	Screening/diagnostic instrument used	Prevalence findings in men and women	Major findings of potential correlates
Håkansson et al. (2018b)	Sweden, athletes (*N* = 352), 60% female, large number of sports	National team level, and conduct college/university (or corresponding) studies	Gaming Addiction Scale, GAS (Lemmens et al., 2009), past 6 months	Problem gaming in 4% of males and 1% of females	Problem gaming significantly associated with problem gambling

## Discussion

The present review paper summarized the current knowledge about prevalence rates and correlates of two of the most well-recognized behavioral addictions in elite athlete populations. A limited number of papers in problem gambling were found, and this preliminary body of evidence demonstrated gender differences with problem gambling rates likely to be higher in men. The evidence remains inconclusive as to whether problem gambling in elite athletes is more common than in the general population, and whether the prevalence differs across different sports. Several studies are characterized by a sparsity in the data and possible associations to study. For problem gaming, the literature search indicated a large need for prevalence studies in this population, as only one paper could be found.

### Problem Gambling—Preliminary Evidence, Gaps, and Recommendations

In five of the studies included about problem gambling, authors concluded that the rates of problem gambling detected in each setting are elevated compared to that of the general population (Håkansson et al., 2018b; Jensen et al., 2019; Rhind et al., 2014; Vinberg et al., 2020). In contrast, the findings from elite level athletes in Australia point in the opposite direction. It should be noted that this was reported only as the percentage with no risk gambling (the non-risk category of the four-step classification used from the Problem Gambling Severity Index, Wynne & Ferris, 2001), that is, without specification of the actual levels of low-risk, moderate-risk, or problem gambling, respectively. However, the difference in non-risk gambling was considerable, such that this paper does not support the overall over-representation of problem gambling in athletes but rather suggests that these associations may vary regionally. Here, more research with scientifically sound assessment, from different geographical regions, is needed, in order to examine whether problematic gambling behaviors are more prevalent in elite athletes. In the general research literature assessing the prevalence of problem gambling, it has been reported that populations with mental health disorders including substance use disorders are at higher risk of reporting problem gambling or being diagnosed with the disorder (Cowlishaw et al., 2014; Dowling, et al., 2015). Apart from that specific risk group, few prevalence measures of problem gambling in specific sub-groups are available, except the general reporting that prevalence figures differ across continents and nations, and across gender and age groups (Calado & Griffiths, 2016). Authors have argued that professional occupation (i.e., one’s job or livelihood), including a link to the world of sports (Vinberg et al., 2021), may be a risk factor of problem gambling (Binde & Romild, 2020). However, prevalence figures in other occupations other than that of athletes are few. The differences reported between nations and continents (Calado & Griffiths, 2016) further indicate the need to repeat prevalence measures in a number of different geographical settings, where the prevalence in the general population can be assumed to differ.

Despite the low number of studies available, the most robust finding—whenever possible to assess from the studies—appeared to be the marked gender difference in problem gambling. The gender differences in problem gambling are well-known from the general population and from clinical populations (Blanco et al., 2006; Calado & Griffiths, 2016; Ekholm et al., 2014; Håkansson et al., 2017). Whenever data in the present studies were reported for each gender separately (Håkansson et al., 2018b, 2020b; Pensgaard et al., 2021; Rhind et al., 2014; Vinberg et al., 2020), these gender differences were confirmed, and it can even be argued that the difference in problem gambling prevalence between men and women may be larger in athletes than in the general population. In Purcell et al.’s study in Australia, no gender difference was reported, and other studies included only (Jensen et al., 2019) or almost only (Grall-Bronnec et al., 2016) men. Although assessed in a limited number of studies, the rates of problem gambling in female elite athletes may not differ—or may differ little—from that of the general female population (Abbott et al., 2018).

There may be several explanations for the markedly large gender difference in problem gambling in athletes. Although in one study only, a similar finding regarding a surprisingly large gender difference in the prevalence of problem gambling was seen from a web survey addressing people with an assumingly high interest in sports, that is, individuals with regular exercise habits and who were social media followers of well-known fitness profiles (Håkansson et al., 2020a). First, one possible explanation may be the fact that women tend to develop gambling problems at a later age than men, such that the assessment of elite athletes, typically young adults, may not detect women who may or may not develop problem gambling later in life, but who have not yet initiated gambling patterns of the same intensity as men. Women have been suggested to have a more rapid evolvement from gambling onset to gambling problems and consistently have shown a higher degree of psychiatric comorbidity in problem gamblers than for their male counterparts (Carneiro et al., 2020; Díez et al., 2014; Granero et al., 2009; Grant et al., 2012; Håkansson et al., 2017; Sundqvist & Rosendahl, 2019). However, this may occur only at a later stage in life than the period during which an individual is typically involved in elite-level sports.

Also, gambling traditionally has been perceived as a primarily stereotyped “male” activity, and although there has been a “feminization” of the gambling market in recent years, with data indicating a narrowing of the gap in prevalence rates (Abbott et al., 2018; Svensson & Romild, 2014), there may still be attitudes and perceptions about gambling, which contribute to the marked gender difference within the world of sports. Male sports, typically some of the major team sports attracting massive media attention, generally involve more money and closer ties to the sports betting world than female sports. Professional male athletes usually make more money than women athletes across sports, which can be a contributing factor to men engaging in gambling. It is insufficiently assessed in the literature and beyond the scope of the present study to show how attitudes toward gambling affect gambling rates and problem gambling in men and women in sports. However, men and women may gamble for different reasons (Stark et al., 2012), and the positive link between sport-related settings and gambling may not apply to women to the same extent as they do to men. In future preventive and empirical work, female athletes’ gambling may need to be further surveyed, as it cannot be excluded that the increase in female gambling in the general population (Abbott et al., 2018; Svensson & Romild, 2014) may also affect female athletes over time.

Few studies assessed differences in problem gambling between different types of sports. In the studies by Håkansson et al., (2018b) and by Vinberg et al. (2020), no obvious differences were seen between team sports and non-team sports (Håkansson et al., 2018b), or between each of the team sports assessed in the work by Vinberg et al. (2020). Hypothetically, it could be expected that gambling cultures and betting practices may explain some differences between sports, as some specific sports, particularly a number of team sports, receive a much larger attention from the gambling industry. Indeed, higher problem gambling was seen in team sports in the UK study (Rhind et al., 2014). Thus, data on this question remain inconclusive, and more research in different settings may need to assess the occurrence of problem gambling in different sports. Also, if such differences do exist, they are likely to be influenced both by individual characteristics of the athletes and by factors related to attitudes, cultures, regulation, and betting involvement in sports. Also, for the scientific discussion around this topic, data from other data sources with which one can compare, are largely lacking. A major challenge is likely to be the power problem related to the study of a large range of very diverse sports with different circumstances, level of professional involvement, and various gambling and sponsorship roles. Thus, larger studies are required and should optimally include a number of different countries.

In the studies included here, there was no information about the gambling types reported by the study participants. While included subjects were athletes, the gambling practices measured by problem gambling instruments may not necessarily be associated with their own sport or with any sports betting overall. Betting on one’s own game was shown to be associated with problem gambling in the international study by Grall-Bronnec et al. (2016), which is an important finding on which to build, given both the risk of problem gambling in athletes themselves and the risk of match-fixing incidents in the case of gambling on a sporting event that athletes can influence individually. Betting on one’s own games or one’s own sport is known to occur and has been described as a major risk of match-fixing events (Moriconi & de Cima, 2020). However, it cannot be excluded that gambling in athletes may also involve gambling types distinct from sports, such as chance-based games. While this is beyond the subject of the present study, further studies should be more thorough about outlining gamblers’ detailed gambling patterns, and how much their gambling is suspected to be due to institutional factors and attitudes in the specific sport. It is also argued that there is a need for longitudinal and mixed method studies in order to gain a more comprehensive understanding of the development of various types of gambling behavior.

Unfortunately, included studies in this review sparsely assessed association with other types of mental health problems. One of the studies failed to demonstrate an association between problem gambling and treatment sought for mental distress (Håkansson et al., 2018b), but mental health was suggested as a correlate of problem gambling in this study and other studies as well. Mental health problems are associated with problem gambling in the general population and may present one of the possible explanations of an increased problem gambling prevalence in athletes where this has been found. The extent to which mental health problems contribute to problem gambling in this population and how attitudes and other sport environment-related factors may be contributing to issues remain to be studied. An increased risk of problem gambling in elite athletes can be suspected for a number of reasons, such as (a) the overall exposure of sports in gambling and gambling in sports (Deans et al., 2017; Håkansson & Widinghoff, 2019; Hing et al., 2017; Pitt et al., 2016), (b) sport sponsorship (Maher et al., 2006), and (c) personality traits common in sports that are likely to contribute to gambling behavior, particularly competitiveness (Harris et al., 2015).

Two of the studies included in the present review were carried out with respect to behavioral changes or changes in mental health in athletes during the COVID-19 pandemic (Håkansson et al., 2020b; Pensgaard et al., 2021). Specifically, the Swedish study was conducted when high-level sports globally were in virtually complete lockdown (Håkansson et al., 2020b), whereas the Norwegian study inclusion period was somewhat longer but still primarily involved the earlier phase of the pandemic (Pensgaard et al., 2021). The specific effects of the COVID-19 pandemic on gambling behaviors are beyond the scope of the present review paper, but both studies clearly demonstrated a gender difference in gambling problems, as in a number of other papers here. In addition, the Swedish study, carried out with a methodology similar to a general population study in the same setting (Håkansson, 2020), concluded that the self-reported changes in gambling behaviors during the pandemic were similar to those reported in the general population, that is, with a limited proportion reporting an increase in gambling, but with a significant link between this and gambling problems (Håkansson et al., 2020a). Given the extensive consequences of the pandemic on the world of sports, including career uncertainty and mental distress (Håkansson et al., 2020b), problem gambling within the group of elite athletes may require further research attention, also with respect to the impact of COVID-19.

### Problem Gaming—Preliminary Evidence, Gaps, and Recommendations

Problem gaming was an area with limited research in elite sport, and only one publication was found. It is difficult to generalize or draw conclusions from this paper, including how problem gaming in elite athletes may relate to the general population. The problem gaming scores reported by Håkansson et al., (2018b) were lower than those from the general population web survey carried out in the same geographical setting, although with a general population sample recruited from a market survey company’s panel members (Karlsson et al., 2019). Given the sparsity of general population prevalence data in the area, and the lack of studies so far with athletes, it is not possible to hypothesize whether athletes are at higher risk or not. Thus, this calls for research with respect to the prevalence and correlates of problem gaming, both with elite athletes and other populations.

Also, the more detailed habits of gaming among athletes are largely unknown. Recently, media reports highlighted the possible increase in gaming practices by athletes during the COVID-19 pandemic, due to the cancellation of their sport events (Washington Post, 2020). This type of reporting may suggest a role of gaming during spare time, but scientific data in the area is lacking, and gaming habits, as well as problem gaming in elite athletes, need to be researched in the future. While this is a universal research need, lifestyle changes during the COVID-19 pandemic, including in athletes (Håkansson et al., 2020b), may further strengthen the need for research addressing problem gambling among elite athletes.

## Limitations

The present study has limitations. While it attempted to summarize the literature in a sub-group rarely researched, the scope of the findings is clearly limited by the low number of publications identified. This is particularly the case for problem gaming, for which only one study was available. In addition, the latter is also made up of one of the smallest studies identified here. Furthermore, the aim of the paper was to assess elite athletes specifically, as a number of available research publications, including a review paper summarizing them (Derevensky et al., 2019), have primarily assessed populations of student-athletes. Comparing studies that target elite athletes includes a degree of uncertainty with respect to the unconcise definition for competing at an elite level. For example, athletes’ living conditions may be differ substantially between a person identified as an elite athlete in a smaller, individual sport, compared to a person belonging to a top division in a large team sport and possibly even with a professional status. Thus, more studies, and larger studies, are needed in order to clearly describe the risk of being a problem gambler, or a problem gamer, in different sub-types of sports, and in different sub-groups of athletes with diverse levels of professional status. Moreover, future studies should aim to include both women and men. Although the majority of studies included in this review could be assessed with respect to gender, two of the studies had included only men (Jensen et al., 2019) or almost only men (Grall-Bronnec et al., 2016). Especially, given the assumingly lower prevalence of problem gambling, or problem gaming, among female athletes, future studies should intend to include a sufficient sample of female participants for these estimates to be valid.

## Conclusions

More research is needed in the area of problem gambling and problem gaming in elite athletes. For problem gaming in particular, research is essentially nonexistent, thus further studies, including comparative studies with the general population, are warranted. In problem gambling, some papers indicate a higher prevalence in male elite athletes compared to their female counterparts. However, additional studies are required to confirm this. Also, further research on gambling in different types of sports are needed. In summary, the present review paper calls for increased research in behavioral addictions in elite athletes. Also, given the high comorbidity of addictive behaviors with other mental health disorders, and the fact that such associations were rarely assessed in the studies identified, further research should address this.
